# Retinal Structure and Function in Eyes with Optic Nerve Hypoplasia

**DOI:** 10.1038/srep42480

**Published:** 2017-02-16

**Authors:** Satoshi Katagiri, Sachiko Nishina, Tadashi Yokoi, Masashi Mikami, Yuri Nakayama, Michiko Tanaka, Noriyuki Azuma

**Affiliations:** 1 Department of Ophthalmology and Laboratory for Visual Science, National Center for Child Health and Development, Tokyo, Japan; 2 Department of Ophthalmology, The Jikei University School of Medicine, Tokyo, Japan; 3 Division of Biostatistics, Center for Clinical Research and Development, National Center for Child Health and Development, Tokyo, Japan

## Abstract

We investigated retinal structure and function in eyes with optic nerve hypoplasia (ONH). Twenty-nine eyes of 18 patients with ONH and 21 eyes of 21 control patients were analyzed. Spectral-domain optical coherence tomography (SD-OCT), full-field electroretinography (FF-ERG), and focal macular ERG (FM-ERG) were performed. SD-OCT analysis of the macular region showed significant ganglion cells complex (GCC) thinning nasally and temporally (*P* < 0.05), but the thickness from the inner nuclear layer (INL) to the retinal pigment epithelium (RPE) became thinner only nasally (*P* < 0.05). SD-OCT analysis of the circumpapillary region showed significant thinning in the retinal nerve fiber layer and from the INL to the RPE (*P* < 0.05). The horizontal SD-OCT images showed variable foveal abnormalities. FF-ERG analysis showed significantly reduced amplitudes (*P* < 0.05) and preserved implicit time in the photopic negative response. The amplitudes and implicit times of the other FF-ERG components did not differ significantly. FM-ERG analysis showed significantly reduced amplitudes (*P* < 0.05) but preserved implicit times in all components. The current study showed the change of retinal structure and function in eyes with ONH compared with those with control, representing by decreased retinal ganglion cells (RGCs) and their axons, foveal abnormalities, and preserved peripheral retina except for the RGCs and their axons.

Optic nerve hypoplasia (ONH) is a stationary and congenital anomaly characterized by a small optic nerve head with a yellowish peripapillary halo, the so-called “double-ring” sign^1^,^2^. ONH usually occurs as an isolated entity unilaterally or bilaterally; however, it often is associated with other ocular diseases such as anirida or systemic diseases^3^,^4^. When ONH is associated with pituitary dysfunction and/or midline brain abnormalities, including partial or complete agenesis of the septum pellucidum and/or corpus callosum, it is diagnosed as a clinical syndrome named septo-optic dysplasia^3^. Several etiologies have been reported to cause ONH, including mutations of the *PAX6* gene, which also are the major causes of aniridia; mutations in the *HESX1* and *SOX2* genes, which cause septo-optic dysplasia; and environmental factors such as maternal diabetes mellitus and fetal alcohol syndrome^4^,^5^,^6^,^7^.

The retinal structure in eyes with ONH has been studied using optical coherence tomography (OCT)^8^,^9^,^10^ and histopathologic analysis^2^,^11^,^12^, with the focus on the ganglion cell layer (GCL) and retinal nerve fiber layer (RNFL). Pathological studies have reported a significant reduction in the thicknesses of the GCL and RNFL in eyes with ONH^2^,^11^,^12^, and these findings have been confirmed non-invasively *in vivo* by OCT measurements^8^,^9^,^10^ with excellent reproducibility^13^.

The retinal function in eyes with ONH has been studied using full-field electroretinography (FF-ERG)^14^,^15^,^16^,^17^,^18^. Objective retinal function can be evaluated qualitatively and quantitatively by FF-ERG, because the retinal origins of the ERG components have been identified^19^,^20^,^21^. Previous FF-ERG studies have reported that most patients with ONH have normal responses except for the change in the i-wave in the photopic response^14^,^15^,^16^,^17^,^18^, which is considered to originate from the off-pathway distal to retinal ganglion cells (RGCs)^21^. However, to the best of our knowledge, no study of eyes with ONH has analyzed the photopic negative responses (PhNR), which are considered to originate from the RGCs and their axons^22^,^23^, or the foveal function using focal macular ERG (FM-ERG).

The current study measured and analyzed the detailed retinal structure and function in eyes with ONH using spectral-domain OCT (SD-OCT), FF-ERG, and FM-ERG and focused on the RGCs and their axons and the fovea and clarified the relationship between the retinal structure and function in eyes with ONH.

## Patients and Methods

The institutional review board of the National Center for Child Health and Development approved the protocol of this study, which adhered to the tenets of the Declaration of Helsinki. Informed consent was obtained from the legal guardians of all participants.

**Figure 1:**
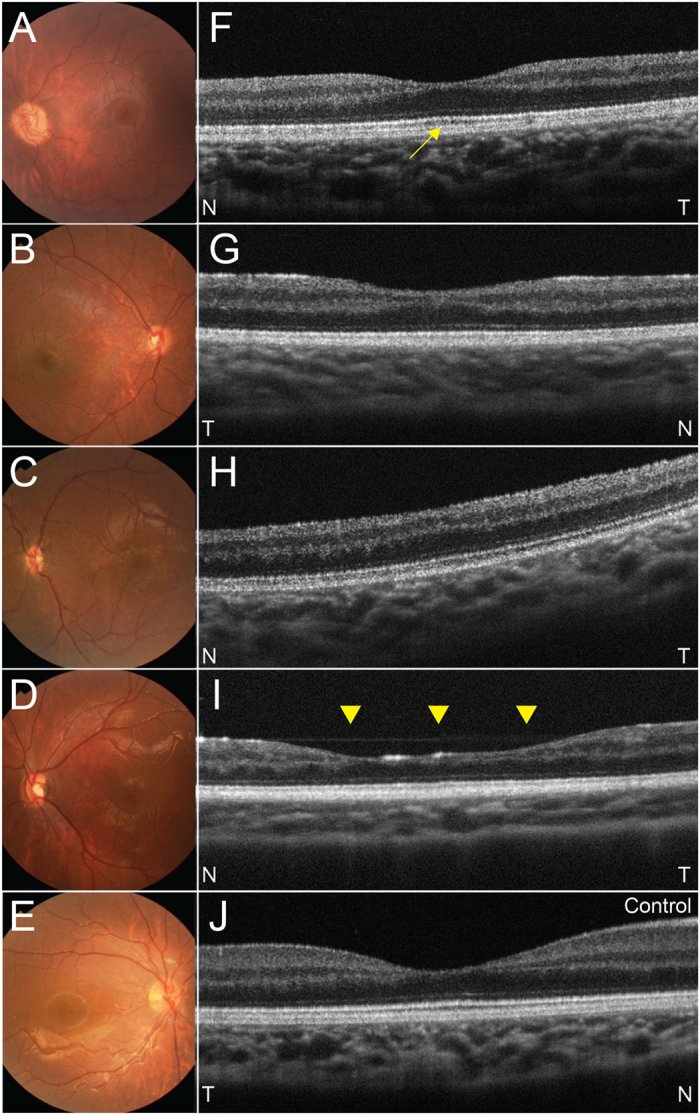
Representative fundus photographs of posterior pole and optical coherence tomography (OCT) images of the fovea in eyes with optic nerve hypoplasia (ONH) and control eyes. (**A**–**D**) Fundus photographs in eyes with ONH. Fundus photographs show that the optic disc sizes are small in all four eyes with under 0.30 of the ratio of horizontal disc diameter to the disc-macula distance. A yellowish peripapillary halo also exists in all four eye. (**E**) fundus photograph of a control right eye. (**F**–**I**) OCT images in eyes with ONH. Each image corresponds to fovea in (**A**–**D**) respectively. (**F**) A left eye with a subnormal fovea. The foveal depression is normal but seems to be shallow due to thinning of the ganglion cell complex. Outer segment lengthening is seen (yellow arrow). (**G)** A right eye with foveal hypoplasia. The foveal depression is incomplete and no outer segment lengthening is apparent. (**H**) A left eye with foveal hypoplasia. No foveal depression or outer segment lengthening is seen. Outer nuclear layer widening is seen. (**I**) A left eye with an atypical foveal abnormality. A foveal depression exists but is wide compared with other types (yellow arrow heads). (**J**) OCT image of a control right eye. T = temporal, N = nasal.

We recruited eyes with ONH and normal eyes as controls from April 2009 to January 2016 in our hospital. Diagnosis of ONH was mainly performed by small disc sizes, that the ratio of the horizontal disc diameter to the disc-macula distance was under 0.30, as previous reported^24^. Other clinical findings such as poor visual acuity, vessel tortuosity, and double-ring sign were used as the assistance of the diagnosis. The diagnosis of ONH was confirmed by the agreements of medical doctors in the current study (S.K., S.N., T.Y., Y.N., M.T., and N.A.). Representative fundus photographs were shown in Fig. 1. Ophthalmic examinations, which were performed under general anesthesia, included a slit-lamp evaluation, dilated funduscopy, SD-OCT, FF-ERG, and FM-ERG. General anesthesia was performed using propofol mainly. We performed the measurements of FF-ERG and FM-ERG continuously just after anaesthetized. The medical records of the patient also were reviewed including the medical and familial histories. Eyes were diagnosed as normal or with ONH depending on the ophthalmic examinations. Patients with optic nerve aplasia, microphthalmos, retinal degeneration, aphakia, and other associated ocular anomalies were excluded. We recruited the control eyes from part of those in previous study^25^. In detail, we recruited the eyes from the previous study, which fulfilled the following three conditions; (1) measured under general anesthesia, (2) examined under 12 years old to match the ages, and (3) performed all the examinations including FM-ERG, FF-ERG, and OCT.

## Patient Demographics

Twenty-nine eyes of 18 patients with ONH (9 girls, 9 boys), and 21 normal eyes of 21 control patients (10 girls, 11 boys) were included. There was no significant (*P* = 0.882) difference in gender between the eyes with ONH and controls. Eleven and seven patients, respectively, had bilateral and unilateral ONH. Six patients were diagnosed with septo-optic dysplasia. No patient had a familial history of ONH or other congenital ocular diseases. The patients ranged in age from 0.5 to 11.3 years (mean ± standard deviation, 4.2 ± 3.1 years); the control subjects ranged in age from 0.3 to 9.1 years (4.3 ± 2.4 years), which did not differ significantly (*P* = 0.934). We did not measure or analyze the visual acuity because the data were considered to be unreliable considering the young patient ages. These data are summarized in Table 1.

## OCT Measurements and Analyses

SD-OCT (RS-3000, Nidek, Gamagori, Japan; software version 1.5.2) was used to measure the retina and analyze the macular and circumpapillary regions. Because we performed all OCT measurements with the patients under general anesthesia and in the supine position with their faces turned to the left, our OCT measurements were rotated 90 degrees to the left compared with those when the images were obtained with a normal head position.

**Figure 2:**
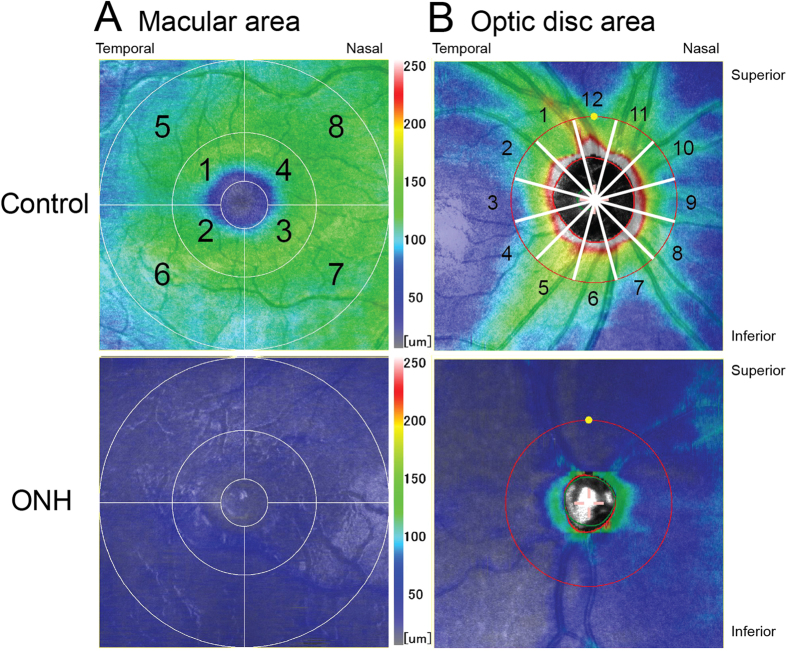
Representative optical coherence tomography (OCT) images of the macular and circumpapillary regions. (**A**) OCT images of the macular region in a control eye and an eye with optic nerve hypoplasia (ONH). The thickness of the ganglion cell complex (GCC) is shown. In the image of the control eye, the eight sectors are numbered. (**B**) OCT images of the circumpapillary region in a control eye and an eye with ONH. The thickness of the retinal nerve fiber layer (RNFL) is shown. In the image of the control eye, the 12 sectors are numbered.

To analyze the macular region, we obtained a 6.0 × 6.0-mm (1,024 A-scans x 64 B-scans) map in which the fovea was centered (we manually corrected the foveal position in some OCT images in which the fovea could not be centered because of foveal hypoplasia) and measured the thickness of the ganglion cell complex (GCC) and that from the inner nuclear layer (INL) to the retinal pigment epithelium (RPE) automatically using the software built into the OCT instrument. Specifically, we measured the thicknesses in the superotemporal, inferotemporal, inferonasal, and superonasal areas for each field 3.0 mm and 6.0 mm in diameter. Each area was numbered from 1 to 8 (Fig. 2A). The number 1 to 8 in the macular region (Fig. 2A) was corresponding to the number 1 to 8 in Table 2.

To analyze the circumpapillary region, we obtained a 6.0 × 6.0-mm map (512 A-scans x 128 B-scans) in which the optic disc was centered and automatically measured the RNFL thickness, the thickness from the INL to the RPE, and the optic disc area using the built-in software. Specifically, we measured the average thicknesses of a global circle around the optic disc; the temporal, superior, nasal, and inferior quadrants; the circumpapillary area separated into 12 30-degree sectors numbered 1 to 12 (Fig. 2B); and the optic disc area. The number 1 to 12 in the circumpapillary area (Fig. 2B) was corresponding to the number 1 to 12 in Table 3.

We used horizontal foveal scans to analyze the foveal structure.

## ERG Measurements and Analyses

FF-ERG was performed as previously described^26^ using white light-emitting diode (LED) built-in contact lens electrodes and a specially designed LED driver (WLS-20, Mayo Co., Inazawa, Japan)^27^. The FF-ERG was recorded using combined recording and analysis systems (Neuropack 8, Nihon Kohden, Tokyo, Japan) with the conditions that adhered to the guidelines of the International Society of Clinical Electrophysiology of Vision^28^.

The FM-ERGs were recorded with Burian-Allen contact lens electrodes (Mayo Co.) with a stimulus spot 15 degrees in diameter for 10 milliseconds using the combined recording and analysis systems (ER80, Kowa Company, Tokyo, Japan, and Neuropack 8).

We measured the following parameters manually: the rod b-wave amplitudes and implicit times, combined rod-cone a- and b-waves, cone a- and b-waves, PhNR, and 30-Hz flicker FF-ERG; and the a- and b-waves amplitudes and implicit times and the PhNR of the FM-ERG.

## Statistical Analyses

The patient demographics were analyzed using the two-tailed and non-paired *t*-tests for the age at examination or chi-square test for gender. Each measurement parameter between eyes with ONH and controls was analyzed using the mixed effect model for repeated measurements following the previous study^29^. The other parameters included the GCC thickness and that from the INL to the RPE in each macular region, optic disc area, RNFL thickness and that from the INL to the RPE in each circumpapillary region, and each component of the FF-ERG and FM-ERG amplitudes and implicit times. The right and left eyes in same case were used as repeated measurements. Fixed effects were categorical variables of the group and eye (left or right). An unstructured covariance matrix was used to account for correlation in the same case. The least squares mean of each group and its 95% confidence interval (CI) were calculated. The difference in the least squares mean and its 95% CI also were calculated. A sandwich estimator was used to estimate the standard error of the least squares mean. The F test was used to test the statistical significance of the difference. Statistical analyses were performed using PASW Statistics 18 (SPSS Inc., Chicago, IL) and SAS software, version 9.4 (SAS Institute, Cary, NC).

## Results

### OCT Analysis

Analyses of the macular region showed significant (*P* < 0.05) reductions in the thickness of the GCC in all eight areas in eyes with ONH compared with controls (Fig. 2A). Analyses of the thicknesses from the INL to the RPE showed significant (*P* < 0.05) reductions in the superonasal and inferonasal areas in diameters of 3.0 and 6.0 mm; no significant reductions in the superotemporal and inferotemporal areas in the same diameters were seen (Table 2).

Analyses of the circumpapillary region showed a significantly (*P* < 0.05) smaller optic disc area, and significant (*P* < 0.05) reductions in the RNFL thickness and those from the INL to the RPE in all global circles, four quadrants, and 12 sectors in eyes with ONH compared with controls (Fig. 2B and Table 3).

The horizontal images in the eyes with ONH showed three foveal appearances: subnormal fovea, foveal hypoplasia, and an atypical foveal abnormality. Seventeen eyes with ONH had a subnormal fovea, which presented with a normal foveal depression that seemed shallow due to thinning of the GCC around the fovea (Fig. 1F) compared with controls (Fig. 1J). Seven eyes with ONH had foveal hypoplasia, which was consistent with the OCT findings previously reported (Fig. 1G,H)^30^. Five eyes with ONH had an atypical foveal abnormality, i.e., a foveal depression was present, but the depression was wider and irregular (Fig. 1I) compared with the other types.

### ERG Analysis

Analyses of the FF-ERG amplitudes showed significant (*P* < 0.05 for all comparisons) reductions of the PhNR in eyes with ONH compared with controls. There were no significant differences in the amplitudes of the other components between eyes with ONH and controls or in the implicit times of all components including the PhNR between eyes with ONH and controls.

Analyses of the FM-ERG amplitudes showed significant (*P* < 0.05 for all comparisons) reductions of all components (a-wave, b-wave, and PhNR) in eyes with ONH compared with controls. There were no significant differences in the implicit times of all components between eyes with ONH and controls.

**Figure 3:**
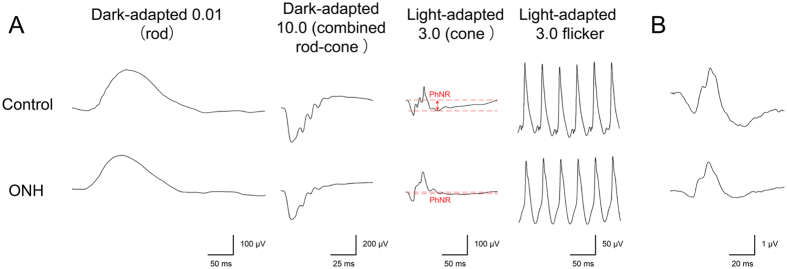
Representative electroretinography (ERG) data from a control eye and an eye with optic nerve hypoplasia (ONH). (**A**) A full-field ERG in an eye with ONH shows normal amplitudes and implicit times in almost all components. Only the amplitude of the photopic negative response (PhNR) is decreased in an eye with ONH (red dash lines and arrow). (**B**) A focal macular ERG in an eye with ONH shows decreased amplitudes but preserved implicit times in all components of the a-wave, b-wave, and PhNR. ms = milliseconds.

Representative FF-ERG and FM-ERG recordings from a normal control eye and an eye with ONH are shown in Fig. 3. The FF-ERG and FM-ERG amplitudes and implicit times are shown in Table 4.

## Discussion

The retinal structure in eyes with ONH has been studied using OCT^8^,^9^,^10^ and histopathologic data^2^,^11^,^12^, and the function has been studied using FF-ERG^14^,^15^,^16^,^17^,^18^; however, these studies have been performed independently. In the current study, we performed SD-OCT, FF-ERG, and FM-ERG in the same eyes with ONH and investigated the retinal structure and function in those eyes.

The OCT analysis showed significant thinning of the GCC in the macular region and the RNFL in the optic nerve region, which agreed with previous studies^8^,^9^,^10^. In addition, the current FF-ERG results showed significant reductions in the PhNR amplitudes but normal implicit times in eyes with ONH and no significant difference in the i-wave amplitude and implicit time between eyes with ONH and controls. Although no other study has analyzed the PhNR in eyes with ONH, a previous study reported a significantly reduced i-wave amplitude and prolonged its implicit time in eyes with ONH of children compared with healthy eyes of adults^15^. In contrast, the prominent i-wave was reported previously in eyes with diseases in which the PhNR is decreased such as primary open-angle glaucoma and optic neuropathy^21^,^23^. The origin of the i-wave is considered to be the off-pathway distal to the RGCs based on an experiment of pharmacologic blockade in monkey retina^21^ and that of the PhNR is considered to be the RGCs and their axons^22^,^23^. The findings regarding the PhNR and i-wave in the photopic response in the current study agreed with the thinning of the GCC and RNFL in the OCT analyses in the current and previous studies^8^,^9^,^15^.

In the current study, the structural analysis of the retinal thickness from the INL to the RPE using OCT showed a significant reduction from the circumpapillary region to the nasal side of the macular region, whereas no significant reduction in the temporal side of the macular region was seen, which agreed with a previous study, except for the significant thinning of the inner segment layer in the temporal macular region^8^. Because structural analysis of the peripheral retina is difficult due to OCT limitations, the extent of the retinal thinning from the INL to the RPE remains unclear in the nasal, superior, and inferior peripheral retina. In contrast, our FF-ERG results, which were within the normal ranges of amplitudes and implicit times of all components except for the PhNR, suggested that the retinal function except for that of the RGCs and axons was in the normal range in eyes with ONH, all of which agreed with previous FF-ERG findings^14^,^15^,^16^,^17^,^18^.

There are likely two areas of thinning: a broad area including the peripheral retina, such as the area of nasal hemiretina with crossed nerve fibers, and a limited area including the circumpapillary region and nasal side of the macular region. If thinning of the retinal thickness from the INL to the RPE ranged across a broad area including the peripheral retina, it would contradict our FF-ERG results, which showed normal amplitudes and implicit times of all components except for the PhNR. Taken together with these findings, in eyes with ONH, the abnormalities except for the RGCs and their axons might exist in a limited area including the circumpapillary region and nasal side of the macular region, and both the structure and function might be preserved in the normal range in the peripheral retina except for the numbers of the RGCs and axons. Further structural study of peripheral retina with the development of OCT will progress our findings of preserved peripheral retina except for RGCs and their axons.

Previous studies have reported that the structure of foveal hypoplasia in ONH is similar to isolated foveal hypoplasia and that in other ocular diseases such as aniridia, albinism, and achromatopsia, except for the thinning of the GCC, which also occurs in foveal hypoplasia in ONH^8^,^30^. In the current study, varying foveal appearances were seen including subnormal fovea, foveal hypoplasia as previous study^30^, and atypical foveal abnormality with apparently wide and irregular foveal depression (Fig. 1). In contrast, the current FM-ERG showed significant reductions in the amplitudes in all components, whereas the implicit times were within the normal range in all components compared with controls. This cannot be explained completely by the retinal thickness in the macular region, and might reflect varying foveal abnormalities including foveal hypoplasia. The mechanism of the foveal hypoplasia in eyes with ONH remains unknown; however, the foveal abnormalities in eyes with ONH might occur secondary to ONH or originate from the same etiology as ONH, because the varying abnormalities occurred (Fig. 1) and foveal development begins after the optic nerve, RGCs, and their axons formed. When we evaluate the vision in eyes with ONH, it is necessary to evaluate foveal abnormalities in addition to the evaluation of the optic nerve head, RGCs, and RNFL.

Multiple etiologies for ONH have been reported, such as mutations in the *PAX6, HESX1*, and *SOX2* genes or environmental factors such as maternal diabetes mellitus and fetal alcohol syndrome^4^,^5^,^6^,^7^. Despite the known etiologies, the mechanism of ONH remains unclear; however, two possibilities are malformation of the optic nerve, RGCs, and their axons or malformation after development of the optic nerve, RGCs, and their axons^8^,^14^,^31^. The mechanism of ONH may differ with each etiology; however, we could not identify genetic and environmental causes of ONH in the current eyes. Further study of ONH from known etiologies is necessary to elucidate the mechanisms.

The current study had several limitations; the case selection bias, the effect of general anesthesia and young examined age on the ERG results, and the heterogeneous state of the fellow eyes of the controls.

In conclusion, the current study showed decreased RGCs and their axons, foveal abnormalities, and preserved peripheral retina except for the RGCs in eyes with ONH compared with controls regarding both retinal structure and function using SD-OCT, FF-ERG, and FM-ERG.

## Additional Information

**How to cite this article**: Katagiri, S. *et al*. Retinal Structure and Function in Eyes with Optic Nerve Hypoplasia. *Sci. Rep.* **7**, 42480; doi: 10.1038/srep42480 (2017).

**Publisher's note:** Springer Nature remains neutral with regard to jurisdictional claims in published maps and institutional affiliations.

## Figures and Tables

**Table 1:** Patient Demographics and Optic Disc Area in the Current Study.

	Control	ONH	*P* Value
No. patients	21	18	
No. eyes	21	29	
Age range (mean ± SD) (years)	4.3 ± 2.4	4.2 ± 3.1	0.934
No. Gender			
Female	10	9	0.882
Male	11	9	
No. Cases of bilateral ONH		11	

No. = number; SD = standard deviation; ONH = optic nerve hypoplasia.

**Table 2:** Thickness of macular region using optical coherence tomography.

	Least squares mean (95% Confidence Interval)		Difference of least squares mean (95% Confidence Interval), P value	
	ONH	Controls	ONH–Controls	P value
Thickness of GCC (μm)				
1	53.0 (47.3 to 58.8)	109.6 (106.0 to 113.1)	−56.5 (−63.0 to −50.1)	<0.0001*
2	55.5 (49.2 to 61.9)	106.4 (102.3 to 110.4)	−50.8 (−58.1 to −43.5)	<0.0001*
3	50.0 (45.6 to 54.3)	112.8 (108.4 to 117.2)	−62.8 (−68.9 to −56.8)	<0.0001*
4	49.4 (44.6 to 54.2)	114.0 (111.1 to 116.8)	−64.6 (−70.3 to −58.9)	<0.0001*
5	48.7 (42.9 to 54.5)	94.4 (90.6 to 98.2)	−45.7 (−52.8 to −38.7)	<0.0001*
6	47.3 (40.9 to 53.8)	92.3 (88.1 to 96.4)	−44.9 (−52.6 to −37.3)	<0.0001*
7	44.2 (39.5 to 48.9)	106.4 (102.2 to 110.6)	−62.2 (−68.7 to −55.7)	<0.0001*
8	44.4 (40.4 to 48.5)	109.9 (106.1 to 113.7)	−65.5 (−71.0 to −59.9)	<0.0001*
Thickness from INL to RPE (μm)				
1	210.9 (205.8 to 215.9)	212.3 (206.8 to 217.7)	−1.4 (−8.5 to 5.7)	0.6929
2	207.7 (202.4 to 213.0)	213.6 (209.5 to 217.6)	−5.9 (−12.6 to 0.9)	0.0871
3	206.2 (201.2 to 211.3)	216.9 (213.0 to 220.7)	−10.6 (−17.1 to −4.2)	0.0019*
4	207.2 (202.0 to 212.5)	218.7 (214.4 to 223.0)	−11.5 (−18.4 to −4.5)	0.0020*
5	193.8 (189.4 to 198.2)	198.8 (195.0 to 202.5)	−4.9 (−10.8 to 0.9)	0.0958
6	189.8 (185.0 to 194.7)	195.2 (191.0 to 199.3)	−5.3 (−11.9 to 1.2)	0.1079
7	186.1 (181.1 to 191.1)	196.6 (192.1 to 201.1)	−10.5 (−17.4 to −3.7)	0.0037*
8	190.7 (185.8 to 195.6)	203.9 (199.9 to 207.9)	−13.2 (−19.7 to −6.8)	0.0002*

GCC = ganglion cell complex; INL = inner nuclear layer; RPE = retinal pigment epithelium; ONH = optic nerve hypoplasia; *P < 0.05.

**Table 3:** Thickness in Circumpapillary Region Using Optical Coherence Tomography.

	Least squares mean (95% Confidence Interval)		Difference of least squares mean (95% Confidence Interval), P value	
	ONH	Controls	ONH–Controls	P value
Optic disc area (mm^2^)	0.93 (0.68 to 1.17)	2.50 (2.33 to 2.67)	−1.57 (−1.86 to −1.29)	<0.0001*
Thickness of RNFL (μm)				
Global	42.3 (39.2 to 45.4)	100.3 (95.0 to 105.5)	−58.0 (−64.1 to −51.8)	<0.0001*
Temporal	37.8 (36.2 to 39.5)	73.9 (70.4 to 77.3)	−36.1 (−39.7 to −32.4)	<0.0001*
Superior	47.7 (42.8 to 52.7)	127.9 (115.9 to 139.9)	−80.2 (−93.1 to −67.3)	<0.0001*
Nasal	37.8 (34.4 to 41.1)	72.2 (65.6 to 78.8)	−34.5 (−41.8 to −27.1)	<0.0001*
Inferior	45.5 (39.0 to 52.1)	128.9 (122.3 to 135.5)	−83.4 (−92.5 to −74.2)	<0.0001*
1	51.6 (40.7 to 62.4)	138.9 (126.6 to 151.2)	−87.3 (−103.1 to −71.5)	<0.0001*
2	38.9 (35.4 to 42.5)	88.1 (80.5 to 95.7)	−49.2 (−57.3 to −41.2)	<0.0001*
3	33.7 (30.4 to 37.0)	64.3 (59.0 to 69.6)	−30.6 (−36.7 to −24.5)	<0.0001*
4	40.0 (36.1 to 43.9)	75.9 (70.6 to 81.3)	−36.0 (−42.3 to −29.6)	<0.0001*
5	50.2 (39.2 to 61.2)	142.6 (131 to 154.1)	−92.4 (−108.4 to −76.3)	<0.0001*
6	44.8 (36.3 to 53.3)	137.4 (127.6 to 147.3)	−92.6 (−106.1 to −79.2)	<0.0001*
7	44.1 (39.6 to 48.6)	104.0 (96.1 to 112.0)	−59.9 (−69.0 to −50.9)	<0.0001*
8	36.3 (32.6 to 40.0)	67.6 (58.6 to 76.5)	−31.3 (−40.8 to −21.7)	<0.0001*
9	36.6 (33.2 to 40.0)	59.2 (53.1 to 65.3)	−22.6 (−29.7 to −15.6)	<0.0001*
10	38.8 (34.5 to 43.0)	88.3 (77.8 to 98.8)	−49.5 (−60.5 to −38.5)	<0.0001*
11	44.5 (40.1 to 49.0)	118.2 (104.6 to 131.9)	−73.7 (−87.9 to −59.5)	<0.0001*
12	47.7 (42.2 to 53.2)	125.8 (106.1 to 145.5)	−78.1 (−98.8 to −57.4)	<0.0001*
Thickness from INL to RPE (μm)				
Global	146.1 (137.3 to 154.9)	173.6 (168.5 to 178.7)	−27.5 (−37.8 to −17.2)	<0.0001*
Temporal	143.5 (134.1 to 152.9)	175.5 (169.5 to 181.5)	−32.0 (−42.7 to −21.3)	<0.0001*
Superior	148.0 (138.3 to 157.6)	174.5 (169.0 to 179.9)	−26.5 (−37.7 to −15.3)	<0.0001*
Nasal	149.3 (141.0 to 157.7)	175.0 (169.3 to 180.7)	−25.7 (−36.1 to −15.2)	<0.0001*
Inferior	143.6 (135.1 to 152.1)	169.5 (162.2 to 176.8)	−25.9 (−36.9 to −14.8)	<0.0001*
1	144.9 (133.8 to 155.9)	173.3 (166.9 to 179.7)	−28.4 (−39.7 to −17.1)	<0.0001*
2	144.6 (134.3 to 154.9)	174.6 (169.2 to 180.1)	−30.0 (−41.5 to −18.6)	<0.0001*
3	143.4 (133.7 to 153.2)	175.2 (167.5 to 182.9)	−31.8 (−42.8 to −20.7)	<0.0001*
4	142.6 (133.7 to 151.5)	176.8 (170.6 to 183.1)	−34.3 (−45.1 to −23.4)	<0.0001*
5	139.4 (131.2 to 147.6)	166.5 (159.5 to 173.5)	−27.1 (−37.7 to −16.5)	<0.0001*
6	144.5 (135.8 to 153.3)	170.2 (162.3 to 178.1)	−25.7 (−37.2 to −14.2)	<0.0001*
7	146.2 (136.9 to 155.6)	173.1 (164.5 to 181.7)	−26.8 (−39.3 to −14.4)	0.0001*
8	148.4 (140.3 to 156.5)	174.7 (168.5 to 180.9)	−26.3 (−36.8 to −15.8)	<0.0001*
9	149.2 (140.2 to 158.3)	174.0 (168.4 to 179.7)	−24.8 (−35.8 to −13.8)	<0.0001*
10	148.9 (140.7 to 157.0)	175.8 (169.1 to 182.5)	−26.9 (−38.0 to −15.9)	<0.0001*
11	151.2 (140.7 to 161.8)	172.5 (164.4 to 180.6)	−21.3 (−35.0 to −7.5)	0.0034*
12	147.2 (138.6 to 155.9)	178.6 (169.3 to 187.9)	−31.4 (−43.9 to −18.8)	<0.0001*

RNFL = retinal nerve fiber layer; INL = inner nuclear layer; RPE = retinal pigment epithelium; ONH = optic nerve hypoplasia; *P<0.05.

**Table 4:** Amplitudes and Implicit Times in Full-Field and Focal Macular Electroretinography.

	Least squares mean (95% Confidence Interval)		Difference of least squares mean (95% Confidence Interval), P value	
	ONH	Controls	ONH–Controls	P value
Full-field ERG Amplitude (μV)				
Rod b-wave	211.4 (185.1 to 237.8)	201.5 (178.8 to 224.3)	9.9 (−25.4 to 45.2)	0.5727
Combined rod-cone a-wave	385.8 (361.7 to 409.9)	373.1 (352.3 to 393.9)	12.7 (−20.2 to 45.6)	0.4396
Combined rod-cone b-wave	491.2 (451.2 to 531.2)	519.4 (473.1 to 565.6)	−28.2 (−89.8 to 33.5)	0.3604
Cone a-wave	72.9 (53.9 to 91.8)	73.8 (66.9 to 80.7)	−0.9 (−21.2 to 19.3)	0.9266
Cone b-wave	139.9 (120.7 to 159.0)	146 (133.4 to 158.7)	−6.2 (−29.1 to 16.8)	0.5906
i-wave	12.6 (7.8 to 17.5)	11.0 (6.8 to 15.2)	1.6 (−5.0 to 8.2)	0.6210
Photopic negative response	28.9 (17.9 to 39.8)	51.7 (44.9 to 58.5)	−22.9 (−36.0 to −9.7)	0.0011*
30-Hz flicker	166.9 (153.1 to 180.8)	172.2 (159 to 185.4)	−5.2 (−24.3 to 13.8)	0.5806
Full-field ERG Implicit Time (ms)				
Rod b-wave	107.8 (105.1 to 110.6)	113.6 (108.1 to 119.2)	−5.8 (−12.0 to 0.4)	0.0648
Combined rod-cone a-wave	10.6 (10.1 to 11.0)	10.6 (10.2 to 11.0)	0.0 (−0.6 to 0.6)	0.9310
Combined rod-cone b-wave	52.6 (50.8 to 54.5)	50.8 (47.9 to 53.7)	1.8 (−1.6 to 5.3)	0.2866
Cone a-wave	15.8 (15.3 to 16.3)	16.0 (15.7 to 16.3)	−0.2 (−0.8 to 0.4)	0.4460
Cone b-wave	36.9 (35.8 to 38.1)	38.3 (36.2 to 40.5)	−1.4 (−3.8 to 0.9)	0.2319
i-wave	56.5 (54.4 to 58.5)	56.1 (53.9 to 58.2)	0.4 (−2.6 to 3.4)	0.7866
Photopic negative response	66.8 (65.2 to 68.3)	65.7 (64.0 to 67.4)	1.0 (−1.2 to 3.3)	0.3626
30-Hz flicker	30.0 (29.0 to 30.9)	29.6 (28.8 to 30.3)	0.4 (−0.8 to 1.6)	0.5081
Focal mauclar ERG Amplitude (μV)				
a-wave	0.58 (0.48 to 0.68)	0.99 (0.87 to 1.11)	−0.41 (−0.56 to −0.26)	<0.0001*
b-wave	1.85 (1.56 to 2.13)	2.51 (2.19 to 2.84)	−0.67 (−1.08 to −0.25)	0.0026*
Photopic negative response	0.54 (0.43 to 0.65)	1.32 (1.08 to 1.55)	−0.78 (−1.03 to −0.52)	<0.0001*
Focal macular ERG Implicit Time (ms)				
a-wave	22.4 (21.9 to 22.9)	23.1 (22.6 to 23.5)	−0.6 (−1.3 to 0.1)	0.0701
b-wave	37.8 (37.1 to 38.4)	38.5 (37.5 to 39.4)	−0.7 (−1.8 to 0.4)	0.2009
Photopic negative response	64.4 (62.3 to 66.5)	64.9 (62.8 to 66.9)	−0.4 (−3.4 to 2.6)	0.7668

ERG = electroretinography; ONH = optic nerve hypoplasia; ms = millisecond; *P < 0.05.
